# Complement activation in secondary thrombotic microangiopathies

**DOI:** 10.1093/ndt/gfaf091

**Published:** 2025-05-15

**Authors:** Johann Morelle, Fernando Caravaca-Fontan, Fadi Fakhouri, Eleni Frangou, Annette Bruchfeld, Jürgen Floege, Safak Mirioglu, Sarah M Moran, Stefanie Steiger, Kate I Stevens, Onno Y K Teng, Selda Aydin, Anuja Java, Sjoerd A M E G Timmermans, Andreas Kronbichler

**Affiliations:** Division of Nephrology, University Hospitals Namur (CHU UCL Namur), Namur, Belgium; de Duve Institute, UCLouvain, Brussels, Belgium; Department of Nephrology, Instituto de Investigación Hospital ‘12 de Octubre’ (imas12), Madrid, Spain; Division of Nephrology and Hypertension, CHUV, University of Lausanne, Lausanne, Switzerland; Department of Nephrology, Limassol General Hospital, State Health Services Organization, Limassol, Cyprus; Department of Basic and Clinical Sciences, University of Nicosia Medical School, Nicosia, Cyprus; Aretaieio Hospital, National and Kapodistrian University of Athens Medical School, Athens, Greece; Department of Health, Medicine and Caring Sciences, Linköping University, Linköping, Sweden; Department of Renal Medicine, Karolinska University Hospital and CLINTEC Karolinska Institutet, Stockholm, Sweden; Division of Nephrology, Rheinisch-Westfälische Technische Hochschule (RWTH) Aachen University Hospital, Aachen, Germany; Department of Immunology, Aziz Sancar Institute of Experimental Medicine, Istanbul University, Istanbul, Turkey; Division of Nephrology, Bezmialem Vakif University Hospital, Istanbul, Turkey; Division of Nephrology, Department of Internal Medicine, Istanbul Faculty of Medicine, Istanbul University, Istanbul, Turkey; Cork University Hospital, University College Cork, Cork, Ireland; Division of Nephrology, Department of Medicine IV, Ludwig-Maximilians-University Hospital Munich, Munich, Germany; Glasgow Renal and Transplant Unit, Queen Elizabeth University Hospital, Glasgow, UK; Center of Expertise for Lupus, Vasculitis and Complement-mediated Systemic disease (LuVaCs), Department of Nephrology, Leiden University Medical Center, Leiden, The Netherlands; Department of Pathology, Cliniques Universitaires Saint-Luc, Brussels, Belgium; Department of Medicine, Division of Nephrology, Washington University School of Medicine, St. Louis, MO, USA; Expert Center for Immune-mediated Kidney Diseases and Vasculitis, Maastricht University Medical Center, Maastricht, The Netherlands; Department of Biochemistry, Cardiovascular Research Institute Maastricht, Maastricht, The Netherlands and; Department of Health, Medicine and Caring Sciences, Linköping University, Linköping, Sweden; Department of Internal Medicine IV, Nephrology and Hypertension, Medical University of Innsbruck, Innsbruck, Austria

**Keywords:** acute kidney injury, complement inhibition, complement system, haemolytic uraemic syndrome, thrombotic microangiopathy

## Abstract

Secondary thrombotic microangiopathies (TMAs) represent a heterogeneous group of diseases associated with a high risk of kidney failure and death despite available therapeutic strategies. Strong evidence implicates complement activation in the pathogenesis of secondary TMA, and emerging data increasingly suggest that pharmacological blockade of the complement improves the outcomes in patients with secondary TMA. Certain forms of secondary TMA, including postpartum TMA, TMA with coexisting hypertensive emergency and *de novo* TMA after kidney transplantation exhibit a high prevalence of pathogenic variants in complement genes, similar to those observed in primary atypical haemolytic uraemic syndrome. These conditions should be considered as complement-mediated TMA triggered by pregnancy or transplantation or in which severe hypertension represents a symptom rather than the aetiology of TMA. Their optimal management relies on early initiation of complement inhibition. Other aetiologies of secondary TMA (i.e. autoimmune diseases, haematopoietic stem cell transplantation, drugs, infections) are typically not linked with complement gene variants and their management primarily focuses on removal of the culprit trigger or treatment of the underlying condition. While well-designed trials are still awaited, a growing body of evidence suggests that complement activation is also involved in the pathophysiology of these diseases. Complement inhibitors, which have been associated with better outcomes, should be considered in patients with severe (life- or organ-threatening TMA) or refractory secondary TMA despite adequate management of the underlying condition. This review summarizes the current understanding and future directions in the management of secondary TMA, emphasizing the potential of complement inhibition as a therapeutic strategy.

## INTRODUCTION: THROMBOTIC MICROANGIOPATHY (TMA) IN ATYPICAL HAEMOLYTIC URAEMIC SYNDROME (aHUS) AND BEYOND

TMA describes a pattern of endothelial injury characterized by pathological alterations within the walls of arterioles and capillaries leading to microvascular thrombosis [1, 2]. In clinical practice, TMA is often associated with the triad of microangiopathic haemolytic anaemia, thrombocytopenia and ischaemic organ injury involving the kidneys or virtually any other organ. TMA may also present as a progressive and isolated increase in serum creatinine in the absence of haematological manifestations, and a kidney biopsy may be required for the diagnosis. The presence of TMA lesions on the kidney biopsy without haematological manifestations is referred to as ‘kidney-limited TMA’ [1, 3, 4].

Conditions causing TMA encompass a wide spectrum of diseases and a better understanding of the underlying mechanisms has led to a pathophysiology-based classification of TMA [1, 2, 5]. The current classification includes thrombotic thrombocytopenic purpura, due to either immune or inherited deficiency in a disintegrin and metalloprotease with thrombospondin type I motif, member 13 (ADAMTS13); Shiga toxin–producing *E. coli*–associated HUS; secondary TMA; and primary aHUS/complement-mediated TMA (hereafter referred to as aHUS), which remains a clinical diagnosis by exclusion of other aetiologies. Despite the significant advances associated with such classification, the current terminology of atypical, primary and secondary HUS is confusing and efforts are being made to propose an updated nomenclature of TMA [2, 6].

aHUS has become a hallmark of complement-mediated diseases and a model for precision medicine, where uncovering the pivotal role of the complement system has driven the development of targeted therapies, significantly enhancing patient outcomes and quality of life. The complement system is a crucial component of the innate immune response, facilitating pathogen elimination through opsonization, inflammation and direct cell lysis. It can be activated via three distinct pathways (classical, lectin and alternative), all of which converge at the formation of C3 convertase. This enzyme cleaves C3 into C3a and C3b, initiating key effector functions: C3a acts as an anaphylatoxin, enhancing inflammation by recruiting and activating immune cells, while C3b promotes opsonization and activates the terminal complement pathway, culminating in assembly of the membrane attack complex and subsequent lysis of the target pathogen. Tight regulation of complement activity is essential to prevent unintended injury to host tissues. This regulation is maintained by both soluble and membrane-bound complement regulatory proteins, including factor H, factor I and membrane cofactor protein.

The discovery in 1999 of abnormalities in the *CFH* gene, which encodes complement factor H, provided the first evidence that dysregulation of the alternative complement pathway plays a central role in the pathogenesis of aHUS/complement-mediated TMA [7, 8]. This pivotal finding was followed by the identification of additional loss-of-function variants in genes encoding other complement regulatory proteins such as *MCP/CD46* and *CFI*, as well as gain-of-function variants in genes encoding key components of the alternative C3 convertase, namely *C3* and *CFB* (reviewed in [8]). Approximately 60% of patients with aHUS harbour either inherited (pathogenic gene variants) or acquired (autoantibodies against factor H) abnormalities affecting the alternative pathway. These defects lead to uncontrolled complement activation, resulting in endothelial injury and the development of TMA (Supplementary Table S1) [1, 2, 7, 8]. aHUS is characterized by incomplete penetrance, indicating that not all family members carrying the same genetic variant will develop TMA. Current hypotheses suggest that additional factors, such as specific risk haplotypes and/or environmental triggers, are necessary to induce TMA in individuals who are genetically predisposed [8]. The interplay between genetics and environmental factors determines disease onset and severity, with individuals carrying stronger genetic predispositions requiring less intense triggers to develop TMA.

The identification of complement dysregulation in aHUS led to the development of eculizumab and ravulizumab, humanized monoclonal antibodies that target complement protein C5, a key component of the terminal complement pathway. By binding to C5, terminal complement inhibitors prevent its cleavage into C5a and C5b, thereby inhibiting the formation of the membrane attack complex (C5b-9). This blockade halts complement-mediated endothelial damage, resulting in normalization of platelet counts, improvement of kidney function and resolution of microangiopathic haemolysis [9–11, 12]. In patients with aHUS, the use of monoclonal antibodies inhibiting the C5 complement protein drastically reduces the progression to kidney failure or death and prevents relapse after kidney transplantation [9–11, 12]. Despite the central role of the complement system, no biomarker with the ability to identify complement dysregulation in the setting of aHUS has been validated for clinical use nor to aid selection of candidates for C5 blockade at the onset of the disease (Supplementary Table S1). Importantly, a normal blood complement profile does not exclude aHUS [2].

In contrast to primary aHUS, secondary TMAs have received relatively little attention over the last decades and now clearly represent an unmet medical need.

## DIAGNOSIS AND MANAGEMENT OF SECONDARY TMA: AN UNMET MEDICAL NEED

**Figure 1:**
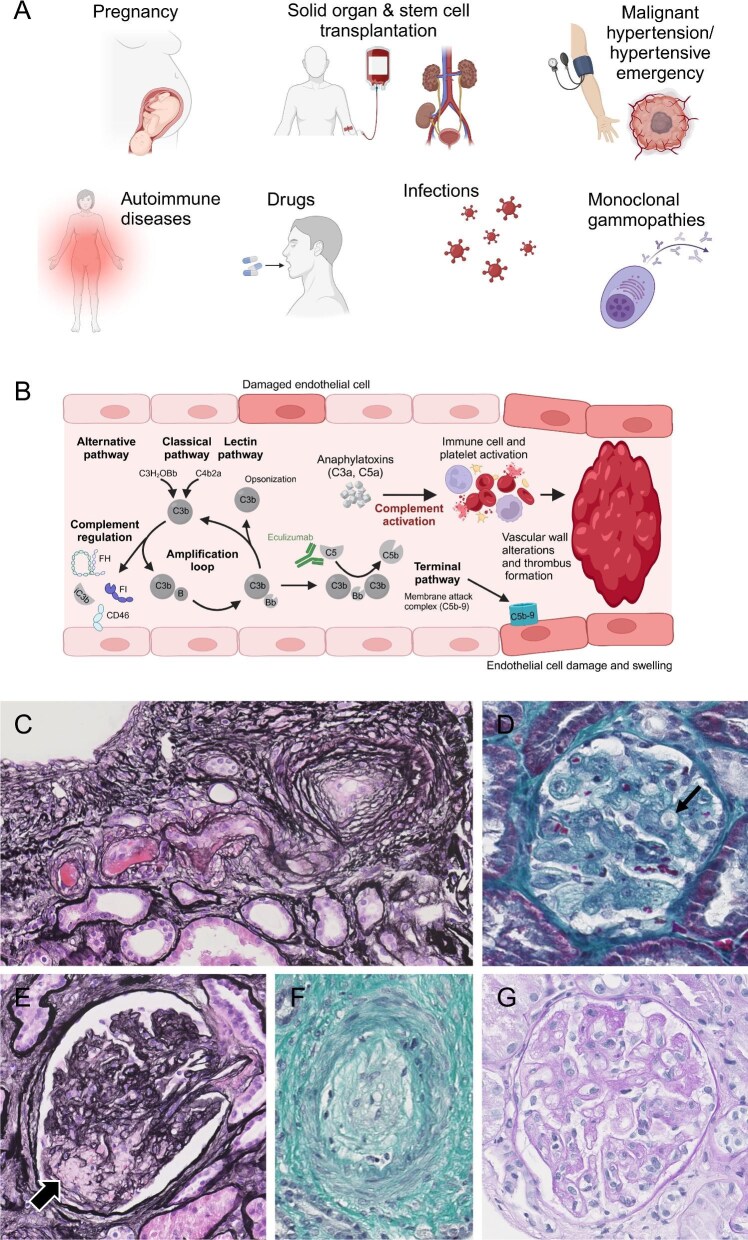
The spectrum of TMAs. **(A)** The most common aetiologies of secondary TMA include pregnancy and postpartum TMA, solid organ and stem cell transplantation, TMA with hypertensive emergency and AKI (‘malignant hypertension’), autoimmune diseases (i.e. SLE, scleroderma renal crisis, APS), drugs, infections and monoclonal gammopathies. **(B)** Activation of the complement system may contribute to endothelial cell damage and to the development of secondary TMA, at least in a subset of patients. **(C-G)** Light microscopy images of kidney biopsies from patients with secondary TMA showing **(C)** arteriolar fibrin thrombi and onion-skin lesions in a patient with TMA and scleroderma renal crisis, **(D)** swelling of glomerular endothelial cells (arrow) in a patient with TMA after HSCT, **(E)** mesangiolysis (arrow) in a patient with drug-induced TMA, **(F)** mucoid changes in the intima of a renal arteriole in a patient with scleroderma renal crisis and **(G)** chronic lesions with double contours of glomerular basement membranes in a patient with TMA after HSCT. Staining was performed using Jones’ silver stain (C and E), Masson's trichrome (D and F) or periodic acid–Schiff (G). Original magnification 20–30×.

Secondary TMAs result from specific diseases or conditions, including pregnancy and the postpartum period, malignant hypertension, solid organ and haematopoietic stem cell transplantation (HSCT), autoimmune diseases or drugs (Fig. 1, Table 1) [1, 2]. The differentiation between aHUS (or complement-mediated TMA) and secondary TMA may sometimes be challenging, as coexisting conditions are found in many patients with complement-mediated TMA, supporting the prevailing hypothesis of a multiple hit process in which a combination of genetic and environmental factors drives TMA [13].

**Table 1:** Aetiologies of ‘secondary TMA’. Modified from Goodship *et al.* [1]. List is not exhaustive.

Pregnancy and postpartum • HELPP syndrome • Pregnancy-associated aHUS/complement-mediated TMA^a^ • APS or other autoimmune diseases
Hypertensive emergency (‘malignant hypertension’) • Shear stress–induced TMA (unrelated to complement) • Complement-mediated TMA/aHUS^a^
Transplantation • Solid organ transplantation: drugs (i.e. CNIs), infection (i.e. CMV), antibody-mediated rejection, *de novo* TMA/complement-mediated TMA^a^ • HSCT: total body irradiation, conditioning regimens, infections, endothelial graft-versus-host disease
Autoimmune diseases • SLE • Scleroderma renal crisis • APS
Drugs • Carfilzomib and other proteasome inhibitors • Gemcitabine, mitomycin C, platinum • VEGF inhibitors • Tyrosine kinase inhibitors • Interferons
Monoclonal gammopathies
Infections • Bacteria: *Streptococcus pneumoniae* • Viruses: HIV, CMV, EBV, influenza, SARS-CoV-2/COVID-19
Cobalamin C defects

a Some TMAs previously referred to as ‘secondary’ are now reclassified as complement-mediated TMA, in which a trigger/condition (postpartum/pregnancy-associated TMA, hypertensive emergency–associated TMA, *de novo* TMA after transplantation) causes TMA in genetically predisposed individuals. Whether other secondary TMAs can be considered as complement-mediated remains to be established.

HIV: human immunodeficiency virus; CMV: cytomegalovirus; EBV: Epstein–Barr virus.

Recent cohort studies highlighted that secondary TMAs represent a significant proportion (49–88%) of all TMAs and are much more common than aHUS (Supplementary Table S2) [5, 14–16, 17]. Despite major advances in the treatment of underlying conditions, secondary TMAs are associated with an unacceptably high risk of progression to kidney failure (33–37%) and death (up to 40%), emphasizing the need for novel, effective therapies (Supplementary Table S2) [5, 16].

The Kidney Disease: Improving Global Outcomes (KDIGO) and International Society of Nephrology included the evaluation of complement inhibitors in secondary TMA among the top priorities in the field of complement-mediated kidney diseases [2, 13]. A phase 3 randomized controlled trial was designed to evaluate the efficacy and safety of ravulizumab, a long-acting complement C5 inhibitor, in adults with secondary TMA (NCT04743804). Unfortunately, the study was terminated prematurely because of continued enrolment challenges. While several ongoing trials are investigating complement inhibitors in secondary TMAs, these studies only cover a small subset of the underlying aetiologies (Table 2).

**Table 2:** List of ongoing clinical trials testing complement inhibitors in secondary TMA.

Condition	Drug	Mechanism	NCT
Hypertensive emergency–TMA	Eculizumab	Anti-C5 monoclonal antibody	NCT05726916
TA-TMA (HSCT)	Ravulizumab	Long-acting anti-C5 monoclonal antibody	NCT04543591
TA-TMA (HSCT)	Pegcetacoplan	C3 inhibitor	NCT05148299
TA-TMA (HSCT)	Nomacopan	Small molecule C5 inhibitor	NCT04784455
TA-TMA (HSCT)	Narsoplimab	Anti-MASP2 monoclonal antibody	NCT05855083
SLE-TMA	KP104	Bifunctional C5 monoclonal antibody factor H fusion protein	NCT05504187
APS nephropathy	RAY121	Inhibitor of classical complement pathway	NCT06371417

MASP2: mannan-binding lectin-associated serine protease 2.

To guide physicians in the management of patients with secondary TMA, we review here the most common aetiologies and discuss their mechanisms, the role of complement and the evidence supporting the use of complement inhibitors for the treatment of these diseases.

## PREGNANCY AND POSTPARTUM-ASSOCIATED TMA

Pregnancy and the postpartum period represent high-risk situations for different forms of TMA and the differential diagnosis can be challenging due to overlapping clinical and laboratory findings. The main aetiologies include aHUS (‘complement-mediated TMA’); haemolysis, elevated liver enzymes and low platelet count (HELLP) syndrome; thrombotic thrombocytopenic purpura and TMA associated with autoimmune diseases (Supplementary Table S3) [18–20].

The postpartum period significantly increases the risk of aHUS due to the heightened activation of the complement system during pregnancy, the abrupt withdrawal of regulatory factors after delivery of the placenta and endothelial stress from delivery-related trauma and/or haemodynamic changes. These triggers can unmask genetic predispositions in complement-regulating proteins, leading to uncontrolled complement activation and complement-mediated TMA. Based on the high prevalence of rare complement gene variants (41–71%; Supplementary Table S4) and clinical features similar to primary aHUS, pregnancy-related aHUS has been reclassified as a complement-mediated TMA [8, 18–22].

Pregnancy-related aHUS occurs in 1 in 25 000 pregnancies and accounts for 7% of all TMA cases [18–20]. Although it may occur during any trimester, pregnancy-related aHUS is the form of TMA that occurs most frequently in the postpartum period (≈80%), and TMA occurring postpartum (up to 3 months after delivery) after an otherwise uneventful pregnancy is highly suggestive of a complement-mediated TMA [18]. Pregnancy and postpartum-related aHUS are associated with a high incidence of severe acute kidney injury (AKI) requiring dialysis (41–71%), a high risk of progression to kidney failure (27–53%) and a significant risk of relapse (28–32%) [21, 22]. Historically, pregnancy-related complement-mediated TMA was managed with therapeutic plasma exchange. However, this was shown to be ineffective in more than half of patients and carried a significant risk of progression to kidney failure. The current standard-of-care treatment of pregnancy-related aHUS relies on the use of complement C5 inhibitors such as eculizumab or ravulizumab, which lead to excellent haematological and renal responses when initiated early [12, 21, 22]. A growing body of evidence supports the safety of eculizumab during pregnancy and lactation [22, 23]. While ravulizumab is currently not recommended during pregnancy due to the lack of safety data, it has been shown to be safe and effective during the postpartum period [12, 24].

HELLP syndrome occurs in ≈0.5–1% of all pregnancies, most often during the third trimester, and represents a severe form of pre-eclampsia [18–20]. The definitive treatment of HELLP syndrome relies on delivery of the foetus and placenta. Persistence of severe thrombocytopenia or kidney dysfunction more than 72 h after delivery should raise suspicion for alternative diagnoses, including aHUS/complement-mediated TMA.

## TMA ASSOCIATED WITH HYPERTENSIVE EMERGENCY (OR MALIGNANT HYPERTENSION)

According to the most recent definitions, hypertensive emergency is defined by blood pressure (BP) ≥180/110 mmHg accompanied by acute hypertension-mediated organ damage, including severe hypertensive retinopathy (i.e. bilateral flame-shaped retinal haemorrhages and/or exudates), microangiopathic haemolytic anaemia and thrombocytopenia, and ischaemic organ damage of the kidney, heart and/or brain [25].

Historically, malignant hypertension was considered to be a complication of high BP causing shear stress to the endothelium with subsequent TMA and AKI. A growing body of evidence supports the notion that TMA associated with hypertensive emergency and AKI actually results from primary defects in complement regulation and complement-mediated TMA [26–31]. Indeed, TMA with coexisting hypertensive emergency is associated with a high prevalence (37–66%) of rare complement gene variants (Supplementary Table S4); evidence of complement activation *in vivo* and *ex vivo* (using a serum-based microvascular endothelial cell assay); a high risk of progression to kidney failure and recurrence of TMA after kidney transplantation; and significant improvement after eculizumab [26–31]. Conversely, systematic evaluation of a large cohort of patients with aHUS showed that severe (grade 2 or 3) hypertension and hypertensive retinopathy (grade 3 or 4) are common in complement-mediated TMA and are reported in 65% and 53% of patients, respectively [30]. Among patients with hypertensive emergencies caused by diseases other than aHUS, the prevalence of TMA is very low (5%) [30]. Importantly, many patients with malignant hypertension caused by complement defects lack features of microangiopathic haemolytic anaemia and/or thrombocytopenia and the diagnosis of TMA requires a kidney biopsy [3, 4, 27, 29].

Early identification of complement-mediated TMA versus TMA resulting from shear stress (caused by hypertension itself) is critical to the optimal care of patients. Neither clinical manifestations, including severe hypertensive retinopathy, nor pathological features on kidney biopsy can differentiate one from the other [29]. In clinical practice, when a patient presents with TMA associated with a hypertensive emergency, BP control should be started immediately, along with a systematic workup for TMA. In patients who fail to improve signs of TMA (including both normalization of platelet level and significant improvement in kidney function) despite adequate BP control (e.g. 3–5 days), initiation of a complement inhibitor should be considered unless there is extensive, irreversible kidney damage (small kidneys on ultrasound, severe interstitial fibrosis/tubular atrophy on kidney biopsy) with limited potential for recovery. As in other forms of aHUS, the use of therapeutic plasma exchange alone is associated with a high risk of kidney failure (>50%), while eculizumab use has been suggested, based on case series, to improve long-term kidney survival (85% at 5 years) [30].

HYPERSHU (NCT05726916) is a phase 3, open-label, randomized controlled trial currently assessing the benefit of complement inhibition in TMA associated with hypertensive emergency and severe AKI (Table 2). In this study, patients with no other condition known to cause TMA (i.e. pregnancy, autoimmunity, drugs, infections) are randomized to receive either BP control alone or with the addition of eculizumab for 3 months. The primary endpoint is kidney function at 6 months.

## TMA AFTER SOLID ORGAN TRANSPLANTATION

Solid organ transplantation, particularly kidney transplantation, represents a significant risk for TMA. This is due to multiple triggers that contribute to the generation of an endothelial-damaging milieu, including ischaemia–reperfusion injury, the use of calcineurin inhibitors (CNIs), infections and rejection [32].

The differential diagnosis of TMA in transplant recipients is broad and includes drug-induced TMA (e.g. CNIs), infections (e.g. cytomegalovirus infection, Shiga toxin TMA), antibody-mediated rejection, recurrence of aHUS after kidney transplantation and *de novo* TMA.

Among patients with kidney failure due to aHUS, the risk of disease recurrence after kidney transplantation has historically been high, ≈60%, with graft loss occurring in ≈90% of those cases. The likelihood of recurrence is strongly influenced by complement genetics, the presence of anti-CFH autoantibodies and any history of recurrence in a previous allograft [33]. Patients carrying pathogenic variants in *CFH, C3* or *CFB* or those who experienced recurrence in a prior transplant are considered at high risk, with recurrence rates ranging from 50% to 100%. Individuals with pathogenic variants in *CFI*, those without identifiable genetic defects or those positive for anti-CFH antibodies are at moderate risk of recurrence. Patients harbouring pathogenic variants in *MCP* or *DGKE*, as well as those with persistently low levels of anti-CFH antibodies, have a low risk of recurrence, estimated to be <10%. Current evidence and international guidelines support the use of prophylactic eculizumab in patients with aHUS who are at high or moderate risk of recurrence, as this treatment has been shown to significantly reduce TMA recurrence and improve graft survival [1, 2, 11].

*De novo* TMA describes a first-onset TMA episode occurring after transplantation in patients without a prior history of aHUS. Post-transplant *de novo* TMA affects 1–10% of kidney transplant recipients and is often triggered by factors such as drug toxicity (e.g. CNIs), infections, antibody-mediated rejection or ischaemia–reperfusion injury [32]. Emerging evidence suggests that a subset of cases may represent complement-mediated TMA, with ≈30% of patients harbouring rare pathogenic variants in complement genes (e.g. *CFH, CFI, CD46*) [33]. Given the potential role of complement dysregulation, early use of complement inhibitors should be considered in *de novo* TMA, particularly when no alternative cause (e.g. infection, drug toxicity) is identified and/or when TMA is severe, persistent or refractory to conventional management [2, 34].

Interestingly, complement inhibition with eculizumab was also successfully used in some patients with TMA associated with antibody-mediated rejection [35, 36] and was suggested to prevent antibody-mediated rejection in kidney recipients with preformed donor-specific antibodies [37] and in recipients sensitized to their living-donor kidney transplants [38], as well as for the management of TMA after ABO-incompatible kidney transplantation [39].

## TMA AFTER HSCT OR TRANSPLANT-ASSOCIATED TMA

Transplant-associated TMA (TA-TMA) represents a major complication of HSCT [40]. Its incidence varies widely across studies and is estimated to be ≈15–20% after allogeneic HSCT [41, 42]. TA-TMA is associated with a significant burden, including kidney failure, and the risk of death from causes other than relapse of the primary disease is higher among patients with TMA [40–42].

In TA-TMA, endothelial injury may result from a combination of factors, including total body irradiation, chemotherapy, CNIs, infections and/or graft-versus-host disease [40]. The pathophysiology of the disease is complex and some have suggested that TA-TMA might be an endothelial manifestation of graft-versus-host disease [40]. The diagnosis of TA-TMA is particularly challenging and can occur months or even years after bone marrow transplantation, often without haematological signs of TMA, and sometimes in the absence of overt graft-versus-host disease. Consequently, clinicians should maintain a high index of suspicion for TA-TMA in patients who exhibit a gradual increase in serum creatinine level following HSCT. This is especially true when there is no improvement after reducing the dosage of CNIs and after excluding other potential causes, such as infectious or toxic aetiologies.

Several lines of evidence suggest that the complement system is activated and contributes to endothelial injury in TA-TMA. First, the presence of the C4d complement protein in glomerular and peritubular capillaries in patients with TA-TMA suggests an antibody-mediated process and supports complement activation via either the classical or lectin pathway [40, 43]. Second, circulating levels of biomarkers of complement activation are higher in patients with TA-TMA compared with other transplant recipients without TMA [44, 45]. Third, small studies reported the presence of anti-CFH autoantibodies in patients with TA-TMA or suggested potential enrichment in gene variants of the alternative complement pathway in transplant recipients developing TMA [46–49]. It should be noted, however, that current biomarkers do not always reliably indicate complement activation and that not all gene variants identified in those studies are pathogenic.

There is currently no established treatment for TA-TMA. Most clinicians withdraw CNIs and use mycophenolate mofetil or ruxolitinib for the prevention or treatment of graft-versus-host disease. While rituximab and defibrotide have been tested in a very small number of patients, accumulating evidence suggests that complement inhibitors such as eculizumab and narsoplimab may offer potential benefits in TA-TMA [50–59]. In retrospective and prospective studies including patients with high-risk TA-TMA and multiorgan injury, the use of an intensive regimen of eculizumab was associated with improved survival rates at 6–12 months (66–71%) compared with untreated historical controls matched for disease severity (17–18%) [57, 58].

Narsoplimab is a fully human monoclonal antibody that binds to and inhibits mannan-binding lectin associated serine protease-2, the effector enzyme of the lectin pathway and an activator of the coagulation cascade. In a single-arm, open-label pivotal trial, 528 patients with TA-TMA were enrolled to receive intravenous narsoplimab once weekly for 4–8 weeks [59]. The response rate, defined as an improvement in both laboratory TMA markers and organ function, was 61%. The results were consistent across components of the primary endpoint, with improvement in laboratory TMA markers in 61% and improvement in organ function in 74% of patients. One-hundred days after the diagnosis of TMA, the survival rate was 68% and 94% in the whole population and in responders, respectively. Narsoplimab was well tolerated, with no apparent safety signal of concern. Narsoplimab is also being tested in paediatric patients with TA-TMA (NCT05855083).

Additional complement inhibitors are currently under investigation in TA-TMA, including ravulizumab; pegcetacoplan, a C3 inhibitor; and nomacopan, a small molecule C5 inhibitor (Table 2).

## SYSTEMIC LUPUS ERYTHEMATOSUS (SLE)

Lesions of TMA are reported in 3–15% of kidney biopsies from patients with lupus nephritis and are associated with a poor prognosis, including an 80% risk of progression to kidney failure within 5 years [60–63]. SLE-associated TMA involves multiple mechanisms, including lupus activity, antiphospholipid antibodies and complement activation. In patients with active SLE, the formation of immune complexes leads to activation of the classical pathway of the complement system, decreasing serum levels of complement C3 and C4 and increasing the complement split products C3b and C3dg as well as the C3dg:C3 and C3b:C3 ratios, which all correlate with disease activity [64]. A subset of patients with SLE-TMA has circulating anti-CFH autoantibodies and/or variants in complement genes, which may further enhance complement activation [65–67]. Again, not all gene variants reported in those studies can be considered as pathogenic.

The first step in the management of patients with SLE-TMA relies on intensification of conventional immunosuppression with corticosteroids and cyclophosphamide or mycophenolate mofetil to control lupus activity [68]. In some patients, however, immunosuppression alone is not sufficient to suppress vascular endothelial damage. Case reports and small case series reported the successful use of anti-complement therapy in SLE-associated TMA [69–71]. A pooled analysis of 30 patients with SLE-TMA treated with eculizumab showed favourable outcomes in 93% [70]. Of note, KP104, a bifunctional C5 monoclonal antibody factor H fusion protein [72], will soon be tested in TMA associated with SLE in a phase 2 trial (NCT05504187). Limited evidence suggests that therapeutic plasma exchange may be associated with higher remission rates in SLE-TMA [73].

## SCLERODERMA RENAL CRISIS

Scleroderma renal crisis is a life-threatening complication that occurs in ≈10% of patients with systemic sclerosis and is characterized by abrupt onset of hypertension, TMA and AKI [74]. Although the prognosis has improved with the use of angiotensin-converting enzyme inhibitors, 40% of patients still require dialysis and 25% die within 1 year [75].

A few observational studies suggest that activation of the complement system might be involved in the pathophysiology of systemic sclerosis and scleroderma renal crisis: hypocomplementemia is present in ≈15% of patients with systemic sclerosis and associated with disease severity and vascular involvement [76, 77]; C5b-9 deposition was detected in capillaries of skin biopsies of systemic sclerosis patients but not in healthy subjects [78]; C4d deposition was found in renal peritubular capillaries in a subset of systemic sclerosis patients with a poor renal outcome [79]; and serum obtained during scleroderma renal crisis induces C5b-9 deposits on cultured microvascular endothelial cells *ex vivo* [80, 81]. The mechanisms driving complement activation in scleroderma renal crisis remain poorly understood. Current hypotheses suggest classical pathway activation may arise from autoantibodies, immune complexes or haemodynamic shear stress secondary to hypertension [80].

Angiotensin-converting enzyme inhibitors remain the cornerstone of treatment for patients with scleroderma renal crisis. In patients with refractory scleroderma renal crisis despite optimal renin–angiotensin blockade, case reports and case series suggest beneficial effects of complement inhibition using eculizumab, with clinical improvement in >80% of cases [80, 82–84].

## ANTIPHOSPHOLIPID SYNDROME (APS)

APS is a systemic autoimmune disease defined by thrombotic or obstetric events that occur in patients with persistent antiphospholipid antibodies [85–87]. APS can be isolated and referred to as ‘primary’ or as secondary to SLE or other autoimmune conditions. In the setting of APS, TMA can present as a rapid decline in kidney function with variable degrees of haematuria and proteinuria (i.e. APS nephropathy) or as part of catastrophic APS [88–90]. Catastrophic APS is a rare, acute and diffuse thrombotic disease with large vessel thrombosis and TMA involving three or more organ systems, with a 50% mortality rate [88, 90].

Although the pathophysiology of APS remains only partially elucidated, a growing body of evidence supports a central role for complement activation in the development of obstetric and thrombotic complications of APS. In mice, genetic deletion of complement factors or the use of drugs targeting specific complement proteins prevented obstetric and thrombotic complications induced by passive transfer of antiphospholipid antibodies from APS patients, including foetal loss, growth retardation, thrombosis or glomerular TMA [91–98]. Interestingly, the protective effect of heparin in APS pregnancies is partially attributed to the ability to inhibit complement activation by antiphospholipid antibodies [86, 92]. In humans, the level of various complement proteins is increased in the serum of APS patients and complement fragments are detected in affected tissues like the placenta and the kidney, with evidence of complement activation via both the classical and alternative pathways [88, 100–104]. Mechanistically, serum from patients with APS or catastrophic APS, as well as purified anti-β2-glycoprotein 1 antibodies, were found to cause C5b-9 deposition and complement-mediated cell death in a ‘modified Ham test’ using endothelial cells prone to complement activation [105]. In this model, complement activation was completely blocked by an anti-C5 monoclonal antibody [105].

Complement activation in APS is initiated by anti-β2-glycoprotein I antibodies binding to their target antigen, triggering the classical pathway. This response is amplified by the alternative pathway, which generates C3 convertase and perpetuates inflammatory signalling [86]. Critical effector molecules include the anaphylatoxins C3a and C5a, which drive neutrophil recruitment, endothelial dysfunction and tissue factor upregulation, alongside the membrane attack complex (C5b-9) that directly damages cells and promotes thrombosis [86]. Additionally, there is a close connection between complement activation and coagulation, cascades that are intrinsically interconnected, contributing to the development of APS complications [86].

Currently, long-term anticoagulation is the only treatment with established efficacy for prevention of large vessel thrombosis in APS [85, 86, 88]. In contrast, little evidence is available to guide the management of patients with TMA in the setting of APS. The current therapeutic approach to catastrophic APS relies on a combination of systemic anticoagulation, corticosteroids, therapeutic plasma exchange and intravenous immunoglobulins [106]. Current recommendations suggest considering complement inhibition for refractory cases of catastrophic APS, based on the underlying pathophysiology and on case reports or case series [106]. Eculizumab was first used to successfully prevent disease recurrence and graft loss in a patient with kidney failure resulting from catastrophic APS who received a kidney transplant from a living related donor [107]. Since this landmark observation, several case reports and series have shown that eculizumab can be successfully used to treat TMA due to APS, mainly in the setting of refractory catastrophic APS [88, 104, 108–112]. Data from the catastrophic APS registry (which includes 584 patients identified over a 30-year period) showed that among the 39 patients treated with eculizumab, 29 (74%) recovered from the episode of catastrophic APS [112]. Compared with non-responders, those who improved after eculizumab had more severe thrombocytopenia and more commonly microangiopathic haemolytic anaemia before initiation of complement inhibition [110, 112].

Additional prospective data are eagerly anticipated to support the use of complement inhibitors in catastrophic APS—either as a rescue or first-line therapy—and in other complications of APS. The RAINBOW trial (NCT06371417) is an ongoing, phase 1b basket trial that is investigating the safety and preliminary efficacy of RAY121, a novel recycling monoclonal antibody against complement C1s, in adult patients with APS and either APS nephropathy or skin involvement or in patients with other immunological diseases.

## DRUG-INDUCED TMA

Drug-induced TMAs account for ≈10% of all TMAs [113]. Drugs may trigger TMA by various mechanisms, including the development of drug-dependent antibodies, direct endothelial cell toxicity or inhibition of vascular endothelial growth factor (VEGF), which has a major role in the maintenance of the glomerular endothelium (Supplementary Table S5) [113–117]. The development of autoantibodies targeting ADAMTS13 has also been reported in response to some therapies (e.g. ticlopidine, clopidogrel, immune checkpoint inhibitors) and is then referred to as drug-associated thrombotic thrombocytopenic purpura [114].

Well-established aetiologies of drug-induced TMA include chemotherapeutic agents (i.e. gemcitabine, mitomycin C, platinum salts), VEGF inhibitors (either systemic or intra-ocular) and tyrosine kinase inhibitors, proteasome inhibitors, pegylated liposomal doxorubicin, CNIs and immune checkpoint inhibitors (Supplementary Table S5) [114, 116, 117].

Unlike pregnancy- or hypertensive emergency–associated TMA, patients with drug-induced TMA show a low prevalence (<2%) of pathogenic variants in complement genes [114, 118]. Despite the absence of genetic defects, some patients with drug-induced TMA show features suggestive of complement activation. The presence of complement proteins in the glomeruli or capillary walls on kidney biopsy are observed in 59% of kidney biopsy specimens and decreased serum levels of complement proteins (isolated low C3 levels) in 36% of the patients with drug-induced TMA [114]. While complement deposition is common in kidney biopsies from patients with gemcitabine-induced TMA, it is unusual in those with TMA due to VEGF or tyrosine kinase inhibitors [114]. The molecular mechanisms of complement activation in a subset of patients with drug-induced TMA remain unknown.

Withdrawal of the culprit drug often leads to complete resolution of TMA. However, additional therapies may need to be considered in patients who fail to respond or present with life-threatening TMA [119, 120]. In a recent systematic review, a short course of eculizumab (a median of six doses) was used in 69 patients with drug-induced TMA because of the persistence of TMA and kidney dysfunction, with good efficacy (complete TMA recovery, 60%; dialysis discontinuation, 59%; kidney recovery, 80%) and tolerance [120].

## OTHER AETIOLOGIES OF SECONDARY TMA

A retrospective study from the Mayo Clinic showed an unexpectedly high prevalence (14%) of monoclonal gammopathy in patients with TMA, especially in the subset of patients ≥50 years of age (21%) compared with an age-matched reference population (4.2%), suggesting a potential pathogenic mechanism [121]. Data from the French monoclonal gammopathy registry indicated that complement biomarker abnormalities are common, with low C3, normal C4 and high soluble C5b-9 levels in 33%, 100% and 77% of tested patients, respectively, suggesting a contribution of the alternative and terminal complement pathways [122]. This is in line with the observation that the serum or purified monoclonal immunoglobulins obtained from these patients induce massive *ex vivo* C5b9 formation in the endothelium [123]. Only a small proportion of these patients carry complement gene variants [122, 123]. Currently, the optimal management of patients with TMA and monoclonal gammopathy is unknown. Future research should investigate the potential benefit versus harm of complement inhibition and clone-directed therapies in this population [124, 125].

Several infections may trigger TMA (Table 1) and recent evidence suggests that complement activation plays a role in the pathogenesis of infection-associated TMA. Elevated levels of complement anaphylatoxins and soluble C5b-9 have been observed in patients with various infection-induced TMAs, including those associated with severe acute respiratory syndrome coronavirus 2 (SARS-CoV-2) [126–128]. The efficacy of complement inhibitors in infection-associated TMA remains controversial and the risk of exacerbating the underlying infection must be carefully weighed against the potential benefits of controlling complement-mediated damage. It is important to recognize that infections, including coronavirus disease 2019 (COVID-19) and influenza, may trigger the development of aHUS/complement-mediated TMA in genetically susceptible patients. If active TMA persists despite clearance of infection, complement inhibition should be considered, alongside a thorough evaluation for underlying complement system abnormalities.

## COMPLEMENT WORKUP IN SECONDARY TMAs

Based on current knowledge, genetic testing and screening for anti-CFH autoantibodies should be conducted in all patients with pregnancy- or postpartum-associated aHUS, TMA related to hypertensive emergencies and de novo TMA following kidney transplantation. These conditions are associated with a high prevalence of rare or pathogenic variants in complement genes (Supplementary Table S4) and represent distinct phenotypes of complement-mediated TMA.

Genetic screening typically involves a next-generation sequencing panel covering at least five key complement genes—*CFH, CFI, CD46*/*MCP, C3* and *CFB*—in combination with multiplex ligation-dependent probe amplification to detect potential *CFHR1*/*CFH* hybrid genes (Supplementary Table S1). Rare variants of uncertain significance may require additional investigation through functional assays, biomarker analyses or other complementary tests to assess their potential pathogenicity.

The current turnaround time for genetic testing is ≈4–6 weeks. While genetic testing is not required to establish a diagnosis of aHUS or complement-mediated TMA, it provides important prognostic information. Specifically, it helps assess the risk of relapse after discontinuation of complement inhibition, stratify recurrence risk following kidney transplantation and guide decisions regarding the duration of prophylactic terminal complement inhibition.

In contrast, pathogenic complement gene variants are uncommon in patients with secondary TMA driven by other underlying conditions. The utility of routine genetic screening in these cases remains uncertain. However, given the possibility of a genetic predisposition in the presence of strong triggers, genetic testing may be considered—particularly in patients with severe clinical presentations or in those being evaluated for complement inhibition therapy.

## CONCLUSIONS AND PERSPECTIVES

Secondary TMAs represent a heterogeneous group of diseases with significant unmet medical needs because they are associated with a high risk of kidney failure and death despite available therapeutic strategies. Strong evidence implicates complement activation in the pathogenesis of secondary TMA, and emerging data increasingly suggest that pharmacological blockade of the complement improves the outcomes in patients with secondary TMA.

**Figure 2:**
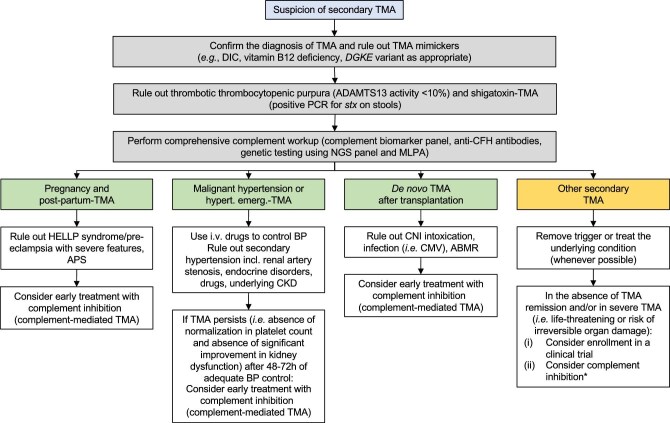
Proposed algorithm for the management of secondary TMA. *Complement inhibition in the setting of secondary TMA is currently off-label. DIC: disseminated intravascular coagulation; PCR: polymerase chain reaction; CFH: complement factor H; NGS: next-generation sequencing; MLPA: multiplex ligation-dependent probe amplification; CMV: cytomegalovirus; ABMR: antibody-mediated rejection.

Certain forms of secondary TMA, including postpartum TMA, TMA with coexisting hypertensive emergency and *de novo* TMA after kidney transplantation exhibit a high prevalence of pathogenic variants in complement genes, similar to those observed in aHUS. These conditions are actually aHUS/complement-mediated TMA triggered by pregnancy or transplantation or in which severe hypertension represents a symptom rather than the aetiology of TMA. Consequently, the optimal management of these diseases relies on early initiation of complement inhibition to improve outcomes (Fig. 2, Table 3).

**Table 3:** Proposed indications for anti-complement therapy in secondary TMA.

Aetiology	Proposed indications for terminal complement inhibitors
Pregnancy-related aHUS	Postpartum- or pregnancy-related TMA in the absence of ADAMTS13 deficiency, antiphospholipid antibodies or other autoimmune disease; or Diagnosis of severe pre-eclampsia or HELLP syndrome that fails to resolve 48–72 h after delivery
Hypertensive emergency–associated TMA	TMA associated with severe hypertension (e.g. systolic BP ≥180 mmHg and/or diastolic BP ≥110 mmHg) and organ damage including hypertensive retinopathy stage 3–4, AKI, acute brain or heart damage; and Absence of TMA recovery (i.e. normalization of platelet level and improvement of kidney function by >25%) despite optimal BP control (e.g. after 3–5 days); and Absence of extensive, irreversible kidney damage (small kidneys on ultrasound, severe interstitial fibrosis/tubular atrophy on kidney biopsy)
*De novo* TMA after transplantation	Absence of an alternative cause (i.e. CMV infection, CNI intoxication, ABMR); and/or Severe (life- or organ-threatening) or non-resolving TMA
TA-TMA	Absence of an alternative cause (i.e. infection, CNI intoxication); and/or Severe (life- or organ-threatening) and/or non-resolving TMA
Lupus-associated TMA	Severe (life- or organ-threatening) and/or non-resolving TMA despite intensification of immunosuppressive treatment
Scleroderma renal crisis	Severe (life- or organ-threatening) and/or non-resolving TMA/scleroderma renal crisis despite optimal angiotensin converting enzyme inhibition and BP control
APS-TMA	Refractory catastrophic APS
Drug-induced TMA	Severe (life- or organ-threatening) or non-resolving TMA despite withdrawal of the culprit drug

CMV: cytomegalovirus; ABMR: antibody-mediated rejection.

Other aetiologies of secondary TMA are typically not linked with pathogenic variants in complement genes. Management primarily focuses on removal of the culprit trigger and the use of complement inhibitors as a rescue therapy, with reports of clinical improvement, even in severe cases. However, publication bias is common and the efficacy of complement inhibitors remains uncertain, highlighting the need for well-designed prospective trials to assess their therapeutic potential. Such studies will require a collaborative effort at a global level including clinicians, pharmaceutical companies, stakeholders and patient associations. Based on the current evidence, we propose to consider the use of complement inhibitors in selected patients, including those with severe (life- or organ-threatening TMA) or refractory TMA despite adequate management of the underlying condition (Fig. 2, Table 3).

The optimal duration of complement inhibitors in secondary TMA is unknown. In the absence of a pathogenic variant in complement genes or anti-CFH antibodies, a short course (8–12 weeks) of complement inhibition might be sufficient in most cases. In the presence of a pathogenic variant or high levels of anti-CFH antibodies, longer treatment durations may be considered on an individual basis, analogous to aHUS [128, 129]. Every patient treated with complement inhibition should be vaccinated with a meningococcal conjugate vaccine covering serogroups A, C, W and Y, as well as a separate vaccine targeting serogroup B, and antibiotic prophylaxis should be considered in accordance with international/national regulatory requirements [1, 2]. When complement inhibition is not available, therapeutic plasma exchange may be considered as an alternative; however, the absence of supporting evidence in secondary TMA and its potential harmful effects should be carefully weighed.

The robustness of evidence linking complement activation to secondary TMA is variable, due in part to the lack of standardized assays and reliable biomarkers to assess and monitor complement activation in clinical practice. Similarly, variability exists in the prevalence of complement gene variants in secondary TMA. Standardized reporting of genetic results should be encouraged, including rare (minor allele frequency <0.01) and pathogenic/likely pathogenic variants using appropriate testing (Supplementary Table S1). Demonstrating enrichment in a specific disease versus the general population is key to support a potential role of genetic drivers in secondary TMA [8].

Perspectives for future research include better identification of patients with secondary TMA who may benefit from complement inhibition, either as a first-line or a rescue therapy; the development and validation of reliable biomarkers and the standardization of functional assays to detect and monitor complement activation; optimization of eculizumab dosing and regimens; evaluation of alternative complement inhibitors and investigation of optimal treatment duration in the various forms of secondary TMA.

This overview highlights the current understanding and future directions in the management of secondary TMA, emphasizing the potential of complement inhibition as a therapeutic strategy.

## Supplementary Material

gfaf091_Supplemental_File

## Data Availability

No new data were generated or analysed in support of this research.
