# Supplementary material for: Complement activation in secondary thrombotic microangiopathies
Source: Nephrol Dial Transplant. 2025 May 15;40(11):2193–206. doi: 10.1093/ndt/gfaf091 (PMC12559796; doi:10.1093/ndt/gfaf091)
Supplement: gfaf091_Supplemental_File [file gfaf091_supplemental_file.docx]

**Activation of the Complement System and the Role of Complement Inhibitors in Secondary Thrombotic Microangiopathies**

Johann Morelle, Fernando Caravaca-Fontan, Fadi Fakhouri, Eleni Frangou, Annette Bruchfeld, Jürgen Floege, Safak Mirioglu, Sarah M. Moran, Stefanie Steiger, Kate I. Stevens, Onno Y.K. Teng, Selda Aydin, Anuja Java, Sjoerd A M E G Timmermans, Andreas Kronbichler;

on behalf of the Immunonephrology Working Group (IWG)
of the European Renal Association (ERA)

**Supplementary Material**

**Table of content**

**Table S1.** Complement workup in atypical HUS/complement-mediated TMA ……………………….2

**Table S2.** Recent cohort studies of secondary TMA ……………………………………………………………….3

**Table S3.** Differential diagnosis and management of TMA associated with pregnancy ………….5

**Table S4.** Prevalence of complement gene variants in the different etiologies of secondary TMA ………………………………………………………………………………………………………………………………………6

**Table S5.** Mechanisms and etiologies of drug-induced TMA ………………………………………………….7

**Table S1. Complement workup in atypical HUS/complement-mediated TMA*.**

| **Genetic testing by next generation sequencing and multiplex ligation-dependent probe amplification** |
| --- |
| - Rare variants in complements genes *CFH, CFI, CD46/MCP, C3, CFB, CFHR1* - Analysis of copy number variation in the *CFH/CFHRs* genomic region - Rare variants in non-complement genes (*DGKE, MMACHC*) - Genotyping for risk haplotypes *CFH*-H3 and *MCP*_ggaac_ |
| **Circulating autoantibodies** |
| - Autoantibodies directed against complement factor H |

*Modified from references (1,2). Currently, anti-CFH autoantibodies are the only biomarkers that significantly influence clinical management during the acute phase of TMA/HUS (1). Other tests - such as plasma complement C3 levels, plasma CFH or CFI concentrations and activity, plasma sC5b-9 concentrations, and CD46 expression on neutrophils - lack sufficient specificity and sensitivity for diagnosing atypical HUS or complement-mediated TMA (2). As a result, they should not be used to differentiate among TMA etiologies or to guide the selection of patients for treatment with terminal complement inhibitors. However, plasma CFH or CFI levels may be useful in evaluating the potential pathogenicity of rare variants of unknown significance in patients with atypical HUS (2).

**Table S2. Recent cohort studies of secondary TMA.** Modified from reference (2).

| **Author, year** | **Le Clech,  2019 (3)** | **Bayer,  2019 (4)** | **Schönermarck,  2019 (5)** | **Henry,  2021 (6)** | **Werion,  2023 (7)** |
| --- | --- | --- | --- | --- | --- |
| **Study characteristics** |  |  |  |  |  |
| Patients with TMA, no. | 110 | 564 | 199 | 216 | 336 |
| Inclusion criteria | Patients with secondary TMA referred for complement analysis | Consecutive patients with TMA | Consecutive patients with TMA | Consecutive patients with TMA | Consecutive patients with TMA |
| Exclusion criteria | SOT, HSCT, C3G, pregnancy-assoc. HUS, hypertensive emergency-TMA | None | None | <18 years | None |
| **Secondary TMA** |  |  |  |  |  |
| Secondary TMA, no. (%) | 110 (100%) | 496 (88%) | 98 (49%) | 188 (87%) | 188 (56%) |
| Main etiologies | Drugs, autoimmune diseases, infections | Pregnancy incl. HELLP, infections, drugs | Cancer, drugs, SOT | HELLP, cancer, HSCT | HSCT, SOT, hypertension |
| Serum creatinine, median, mg/dl | 3.9 | 1.6 | 2.3 | 2.7 | 2.7 |
| Dialysis during hospitalization, % | 41 | 18 | N/A | 21 | 27 |
| Hemoglobin, median, g/dl | 8.7 | 8.3 | N/A | 8.8 | 8.3 |
| Platelets, median, 10^3^/µl | 94 | 63 | 74 | 50 | 77 |
| Neurological involvement, % | 18 | 27 | 36 | 35 | 28 |
| Pathogenic or likely pathogenic variants, no./tested (%) | 2/110 (2%) | N/A | N/A | N/A | 2/65 (3%)* |

*The two patients with pathogenic or likely pathogenic gene variants in complement genes had pregnancy-associated HUS and de novo TMA after kidney transplantation. SOT, solid organ transplantation; HSCT: hematopoietic stem cells transplantation; C3G, C3 glomerulopathy; HELLP : hemolysis, elevated liver enzymes and low platelets syndrome; N/A, not available.

**Table S3. Differential diagnosis and management of TMA associated with pregnancy.**

|  | **Thrombotic thrombocytopenic purpura** | **HELLP** | **Atypical HUS (complement-mediated TMA)** | **Antiphospholipid syndrome** |
| --- | --- | --- | --- | --- |
| **Timing during pregnancy** | Anytime during pregnancy | Mainly during the third trimester | Mainly (80%) during the postpartum; can occur anytime during pregnancy | Anytime during pregnancy |
| **ADAMST13 activity** | <10% | >10% | >10% | >10% |
| **Additional biological features** | Anti-ADAMTS13 IgG (immune) or pathogenic variant in *ADAMTS13* gene (congenital) | LDH >600 IU/L  ALT, AST ≥2 ULN  Platelets <100,000/µl | Pathogenic gene variant in complement genes (50-60%) or anti-CFH antibodies | APL antibodies |
| **Treatment** | Immune: therapeutic plasma exchange, steroids (off-label use of caplacizumab in refractory cases?)  Congenital: fresh frozen plasma or recombinant ADAMTS13 | Delivery of fetus and placenta (resolution of TMA within 48-72 hours after delivery) | Complement inhibition using eculizumab or ravulizumab; if complement inhibitors not available, therapeutic plasma exchange | Depends on previous complications and severity – mainly LMWH and low dose aspirin (more detailed information in the section on APS) |

HELLP, hemolysis, elevated liver enzymes, low platelet count syndrome; HUS, hemolytic uremic syndrome; TMA, thrombotic microangiopathy; ADAMTS13, a disintegrin and metalloproteinase with a thrombospondin type 1 motif, member 13; LDH, lactate dehydrogenase; ALT, alanine aminotransferase; AST, aspartate aminotransferase; ULN, upper limit of normal; CFH, complement factor H; APL, antiphospholipid; LMWH, low-molecular weight heparin; APS, antiphospholipid syndrome.

**Table S4. Prevalence of complement gene variants in the different etiologies of secondary TMA.**

| **Secondary TMA** | **Prevalence of rare/pathogenic complement gene variants** |
| --- | --- |
| Pregnancy or postpartum-associated TMA | 41-71% (8-10) |
| Hypertensive emergency-associated TMA | 37-66% (11-13) |
| *De novo* TMA after kidney transplantation | 29% (14) |
| Other secondary TMAs | <5%* (3,7) |

*In the French cohort, 4 patients out of 110 (3.6%) had a rare variant in relevant complement genes (not considering *THBD*), and 2 (1.8%) had pathogenic variants (3). In the Belgian cohort, rare gene variants and pathogenic or likely pathogenic variants were found in 8/64 (13%) and 2/64 (3%) patients with secondary TMA, respectively (7). The two patients with secondary TMA and pathogenic/likely pathogenic variants had postpartum-associated TMA and de novo TMA after kidney transplantation (7).

**Table S5. Mechanisms and etiologies of drug-induced TMA.**

|  | **Immune drug-induced TMA,  antibody-dependent** | **Non-immune drug-induced TMA,  direct toxicity** | **Drug-associated thrombotic thrombocytopenic purpura** |
| --- | --- | --- | --- |
| Mechanism | Occurs after exposure to a drug, idiosyncratic, antibody-dependent mechanism (interaction with a naturally occurring antibody that can then bind to a cell surface epitope), sudden onset | Does not involve drug-dependent antibodies; dose-related, progressive disease | Severe ADAMTS13 deficiency due to inhibitory autoantibodies against ADAMTS13; underlying mechanism is uncertain |
| ADAMTS13 activity | >10% | >10% | <10% |
| Etiologies* | Quinine  Antimicrobials: trimethoprim-sulfamethoxazole, ciprofloxacin  Gemcitabine  Adalimumab  Quetiapine | Calcineurin inhibitors  Sirolimus, everolimus  Cancer drugs: gemcitabine, mitomycin C, pegylated liposomal  doxorubicin  Vascular endothelial growth factor inhibitors and tyrosine kinase inhibitors  Proteasome inhibitors: bortezomib, carfilzomib, ixazomib  Interferons  Valproic acid  Cocaine | Immune checkpoint inhibitors: ipilimumab, pembrolizumab, atezolizumab  Anti-platelet agents: ticlopidine, clopidogrel, ticagrelor  Immunosuppressive therapies: infliximab, methotrexate, ustekinumab |

*List is not exhaustive. TMA, thrombotic microangiopathy; ADAMTS13, a disintegrin and metalloproteinase with a thrombospondin type 1 motif.

**Supplementary references**

1. Vivarelli M, Barratt J, Beck LH Jr, et al. The role of complement in kidney disease: conclusions from a Kidney Disease: Improving Global Outcomes (KDIGO) Controversies Conference. Kidney Int. 2024 Sep;106(3):369-391. doi: 10.1016/j.kint.2024.05.015.
2. Fakhouri F, Schwotzer N, Frémeaux-Bacchi V. How I diagnose and treat atypical hemolytic uremic syndrome. Blood. 2023 Mar 2;141(9):984-995. doi: 10.1182/blood.2022017860.
3. Le Clech A, Simon-Tillaux N, Provôt F, et al. Atypical and secondary hemolytic uremic syndromes have a distinct presentation and no common genetic risk factors. Kidney Int. 2019 Jun;95(6):1443-1452. doi: 10.1016/j.kint.2019.01.023.
4. Bayer G, von Tokarski F, Thoreau B, et al. Etiology and Outcomes of Thrombotic Microangiopathies. Clin J Am Soc Nephrol. 2019 Apr 5;14(4):557-566. doi: 10.2215/CJN.11470918.
5. Schönermarck U, Ries W, Schröppel B, et al. Relative incidence of thrombotic thrombocytopenic purpura and haemolytic uraemic syndrome in clinically suspected cases of thrombotic microangiopathy. Clin Kidney J. 2019 Jun 18;13(2):208-216. doi: 10.1093/ckj/sfz066.
6. Henry N, Mellaza C, Fage N, et al. Retrospective and Systematic Analysis of Causes and Outcomes of Thrombotic Microangiopathies in Routine Clinical Practice: An 11-Year Study. Front Med (Lausanne). 2021 Feb 26;8:566678. doi: 10.3389/fmed.2021.566678.
7. Werion A, Storms P, Zizi Y, et al. Epidemiology, Outcomes, and Complement Gene Variants in Secondary Thrombotic Microangiopathies. Clin J Am Soc Nephrol. 2023 Jul 1;18(7):881-891. doi: 10.2215/CJN.0000000000000182.
8. Bruel A, Kavanagh D, Noris M, et al. Hemolytic uremic syndrome in pregnancy and postpartum. Clin J Am Soc Nephrol. 2017;12:1237-1247.
9. Huerta A, Arjona E, Portoles J, et al. A retrospective study of pregnancy-associated atypical hemolytic uremic syndrome. Kidney Int. 2018 Feb;93(2):450-459. doi: 10.1016/j.kint.2017.06.022
10. Gaggl M, Aigner C, Csuka D, et al. Maternal and Fetal Outcomes of Pregnancies in Women with Atypical Hemolytic Uremic Syndrome. J Am Soc Nephrol. 2018 Mar;29(3):1020-1029. doi: 10.1681/ASN.2016090995.
11. Timmermans S, Abdul-Hamid MA, Vanderlocht J, et al. Patients with hypertension-associated thrombotic microangiopathy may present with complement abnormalities. Kidney Int. 2017;91:1420-1425.
12. Cavero T, Arjona E, Soto K, et al. Severe and malignant hypertension are common in primary atypical hemolytic uremic syndrome. Kidney Int. 2019;96:995-1004.
13. El Karoui K, Boudhabhay I, Petitprez F, et al. Impact of hypertensive emergency and rare complement variants on the presentation and outcome of atypical hemolytic uremic syndrome. Haematologica. 2019;104:2501-2511.
14. Le Quintrec M, Lionet A, Kamar N, et al. Complement mutationassociated de novo thrombotic microangiopathy following kidney transplantation. Am J Transplant. 2008;8:1694-1701.
